# e-Learning and Web-Based Tools for Psychosocial Interventions Addressing Neuropsychiatric Symptoms of Dementia During the COVID-19 Pandemic in Tokyo, Japan: Quasi-Experimental Study

**DOI:** 10.2196/30652

**Published:** 2021-10-12

**Authors:** Miharu Nakanishi, Syudo Yamasaki, Kaori Endo, Junko Niimura, Canan Ziylan, Ton J E M Bakker, Eva Granvik, Katarina Nägga, Atsushi Nishida

**Affiliations:** 1 Department of Psychiatric Nursing Tohoku University Graduate School of Medicine Sendai-shi Japan; 2 Research Center for Social Science & Medicine Tokyo Metropolitan Institute of Medical Science Setagaya-ku Japan; 3 Research Center Innovations in Care Rotterdam University of Applied Sciences Rotterdam Netherlands; 4 Stichting Wetenschap Balans Rotterdam Netherlands; 5 Center for Excellence in Dementia University Hospital, Malmö Malmö Sweden; 6 Clinical Memory Research Unit Department of Clinical Sciences Malmö Lund University Malmö Sweden; 7 Department of Acute Internal Medicine and Geriatrics Linköping University Linköping Sweden

**Keywords:** dementia, home care services, implementation science, nursing homes, web-based tool

## Abstract

**Background:**

Concern has been raised that the COVID-19 pandemic and consequent social distancing measures may increase neuropsychiatric symptoms in people with dementia. Thus, we developed and delivered an e-learning training course to professional caregivers on using a web-based tool for psychosocial interventions for people with dementia.

**Objective:**

The aim of our study was to evaluate the feasibility and efficacy of an e-learning course in combination with a web-based tool in addressing neuropsychiatric symptoms of dementia.

**Methods:**

A quasi-experimental design was used in Tokyo, Japan. The e-learning course was delivered three times to professional caregivers between July and December 2020. Caregivers who completed the course assessed the level of neuropsychiatric symptoms in people with dementia using the total score from the Neuropsychiatric Inventory (NPI) via a web-based tool. The primary outcome measures were the number of caregivers who implemented follow-up NPI evaluations by March 2021 and the change in NPI scores from baseline to their most recent follow-up evaluations. As a control group, information was also obtained from professional caregivers who completed a face-to-face training course using the same web-based tool between July 2019 and March 2020.

**Results:**

A total of 268 caregivers completed the e-learning course in 2020. Of the 268 caregivers, 56 (20.9%) underwent follow-up evaluations with 63 persons with dementia. The average NPI score was significantly reduced from baseline (mean 20.4, SD 16.2) to the most recent follow-up evaluations (mean 14.3, SD 13.4). The effect size was assumed to be medium (Cohen *d*_rm [repeated measures]_=0.40). The control group consisted of 252 caregivers who completed a face-to-face training course. Of the 252 caregivers, 114 (45.2%) underwent follow-up evaluations. Compared to the control group, caregivers who completed the e-learning course were significantly less likely to implement follow-up evaluations (*χ*^2^_1_=52.0, *P*<.001). The change in NPI scores did not differ according to the type of training course (baseline-adjusted difference=–0.61, *P*=.69).

**Conclusions:**

The replacement of face-to-face training with e-learning may have provided professionals with an opportunity to participate in the dementia behavior analysis and support enhancement (DEMBASE) program who may not have participated in the program otherwise. Although the program showed equal efficacy in terms of the two training courses, the feasibility was suboptimal with lower implementation levels for those receiving e-learning training. Thus, further strategies should be developed to improve feasibility by providing motivational triggers for implementation and technical support for care professionals. Using online communities in the program should also be investigated.

## Introduction

Dementia is a public health concern since people are living longer and age increases the risk of dementia. Globally, the total number of people with dementia was estimated to be 46.8 million in 2015 and is projected to rise to 131.5 million by 2050 [1]. Dementia is chronic and progressive in nature, caused by a variety of brain illnesses that affect memory, thinking, behavior, and ability to perform everyday activities. It is estimated that 5% to 8% of the general population aged 60 years and over, at any given time, have dementia. Dementia affects individuals, their families, and the economy, with global costs estimated at approximately US $1 trillion annually [2]. Japan also faces an expected increase in the number of people with dementia. This number is estimated to reach 10.2 million by 2050, accounting for 10% of the total population [3].

The COVID-19 pandemic has disproportionately impacted people living with dementia [4]. The severity and mortality of COVID-19 worsens with age [5] and in individuals with pre-existing illnesses, such as hypertension and diabetes [6,7], which are common in people with dementia [8]. Furthermore, people with dementia may not understand or remember the required COVID-19 preventive measures, such as wearing a facial mask, physical distancing, and hygiene, because of their cognitive impairment [9].

Concern has been raised that the COVID-19 pandemic and consequent social distancing measures may increase neuropsychiatric symptoms in people with dementia [10]. Neuropsychiatric symptoms, such as shouting, wandering, agitation, resistance to care, depression, anxiety, apathy, and other behaviors, are considered expressions of distress in people with dementia. Neuropsychiatric symptoms are common in people with dementia both in the community [11] and in nursing homes [12], resulting in higher psychotropic drug use [13] and increased mortality [14]. Women are more likely to exhibit a broader range of symptoms compared to men [15]. Since neuropsychiatric symptoms represent unmet needs and distress, psychosocial interventions are globally recommended as first-line treatments to target the underlying causes [4]. Social distancing measures related to the COVID-19 pandemic, such as home confinement and restrictions on visitors for nursing home residents, imposed a risk for social isolation and/or loneliness and they limited physical activity for people with dementia [16]. This has been shown to negatively impact neuropsychiatric symptoms, such as anxiety and depression [17-19], in people with dementia and to cause further cognitive and functional decline [17,20]. However, some nursing home residents in the Netherlands were reported to have decreased neuropsychiatric symptoms, such as agitation and aggression, due to a reduction in overstimulation [21]. Therefore, monitoring the potential long-lasting effects of COVID-19 on neuropsychiatric symptoms and the effectiveness of interventions delivered remotely through technology is warranted [4,10].

To address neuropsychiatric symptoms, a face-to-face training course was delivered with a web-based tool to professional caregivers who participated in the psychosocial dementia behavior analysis and support enhancement (DEMBASE) program. The results of a cluster-randomized controlled study indicated that DEMBASE is effective in reducing neuropsychiatric symptoms in people with dementia [22]. Based on these results, the Tokyo Metropolitan Government introduced the DEMBASE program into the daily practice of professional caregivers in 2018 [23].

Although the demand for e-learning platforms is emerging among essential care workers, no evaluation exists that tests the feasibility and efficacy of the digital transformation of the psychosocial dementia care program for neuropsychiatric symptoms. Therefore, we developed an e-learning training course for professional caregivers using a web-based tool for psychosocial interventions for people with dementia in Tokyo, Japan. The e-learning course aimed to replace the face-to-face training course to avoid group gatherings during the COVID-19 pandemic in 2020. Furthermore, this study evaluated the feasibility and efficacy of the e-learning course combined with a web-based tool in addressing neuropsychiatric symptoms of dementia. We hypothesized that the e-learning course would sustain the efficacy in reducing neuropsychiatric symptoms because the interventions include discussions with a multidisciplinary discussion team. Feasibility, as measured by the percentage implementation, was anticipated to be lower in the e-learning training course due to reduced availability of human resources during the COVID-19 pandemic.

## Methods

### Design

**Figure 1:**
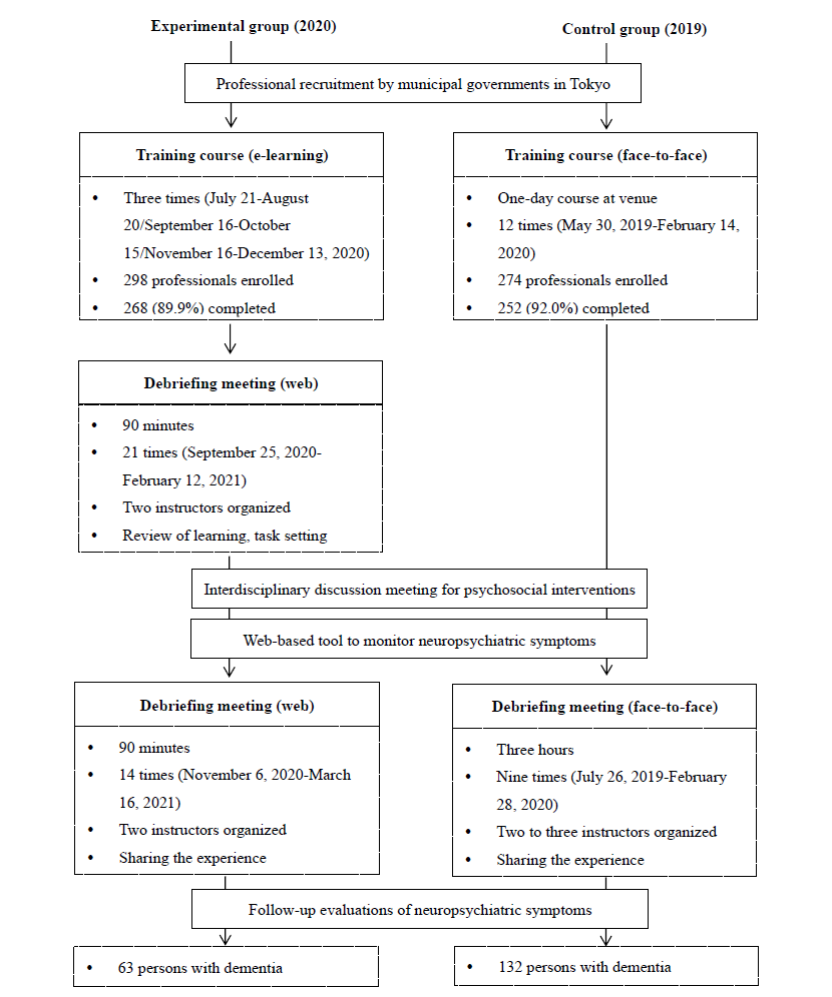
Flow of the psychosocial dementia care program in the experimental (e-learning course) and control (face-to-face training) groups.

A quasi-experimental, longitudinal design was used (Figure 1). The experimental group consisted of 268 professional caregivers who completed the e-learning course and participated in the DEMBASE program between July 2020 and March 2021. The control group consisted of 252 professionals who completed a face-to-face training course and participated in the program between May 2019 and March 2020.

### Procedure

Data were collected in naturalistic long-term care settings from April 2019 to March 2020 for the control group, and from April 2020 to March 2021 for the experimental group. Each municipal government independently decided whether to apply for the fund, which was approved for use in the recruitment of care providers and other professionals and in conducting a training course among all participating professionals.

All professionals were informed of their voluntary participation during the recruitment process, and their application to participate was regarded as consent. Participating professionals acquired informed consent from persons with dementia and/or their family members prior to providing care.

Since the e-learning training course was introduced by the government in response to the COVID-19 pandemic, we could not determine the sampling process and sample size a priori for this study. We anticipated that the rate of implementation would be 15% lower in the experimental group compared to the control group. The rate of implementation in the control group was expected to be equal to that in our previous study (46.4%) [23]. Therefore, the desired sample size of professionals was 283 per group, which was calculated using G*Power software (version 3.1.9.7; Heinrich-Heine-Universität Düsseldorf) [24,25].

### Participants

Care professionals working as long-term service providers were invited to participate in the DEMBASE program. Potential participants included care managers, nurses, and other direct care workers who worked for providers that were accredited by the public long-term care insurance program. We included all the professionals who applied to participate in the program. Participant recruitment was conducted by each municipality that applied to the program. In 2019, 10 municipalities participated in the face-to-face training course. The number of participating municipalities increased to 23 in 2020; this was due to the availability of e-learning.

The approaches for the recruitment process, therefore, varied according to municipality-based choices (eg, an application form on a website, an oral invitation sent to 3-4 providers, and holding a seminar for the directors of the providers). Under the funding rule established by the Tokyo Metropolitan Government, the DEMBASE program was made available for in-home care management agencies, in-home care services, and residential care services. In-home care management agencies handle monthly care plans for in-home care recipients and are independent from the providers of in-home care services. In-home care service providers offer direct care to individuals who live in their houses. Residential care service providers offer a residential care package to individuals who reside in facilities, and they handle the monthly care plans for their care recipients.

### Intervention

#### Overview

The DEMBASE program was comprised of (1) a training course, (2) an interdisciplinary discussion meeting (analysis), (3) a web-based tool for ongoing behavioral assessments (dementia behavior), and (4) a debriefing meeting (support enhancement). The timing of the training course and debriefing meetings varied by municipality (Figure 1). The characteristics of the programs in both groups are summarized in Table 1.

**Table 1:** Characteristics of the dementia behavior analysis and support enhancement (DEMBASE) program in the experimental group (e-learning) and control group (face-to-face training course).

Program characteristic	Experimental group	Control group
Time period	July 21, 2020-March 31, 2021	May 30, 2019-March 31, 2020
Training course details	e-Learning delivery 5.5 hours on average	Face-to-face delivery 1-day course at venue
Interdisciplinary discussion meeting: action items	Evaluation of neuropsychiatric symptoms Specification of unmet needs Establishment of an interdisciplinary action plan	Evaluation of neuropsychiatric symptoms Specification of unmet needs Establishment of an interdisciplinary action plan
Web-based tool: contents	Neuropsychiatric Inventory (NPI) Basic physical needs and environmental sources of discomfort Prescribed medication for the nervous system	Neuropsychiatric Inventory (NPI) Basic physical needs and environmental sources of discomfort Prescribed medication for the nervous system
Debriefing meeting details	Zoom meeting Two meetings, 90 minutes each First meeting: review of learning and setting a task Second meeting: sharing the experience of the program	Face-to-face meeting 1 day, 3 hours at venue Sharing the experience of the program

#### Training Course

##### Overview

The training course guided (1) the process of an interdisciplinary meeting to evaluate neuropsychiatric symptoms, specify unmet needs using a 23-item checklist, and establish an action plan using an interdisciplinary approach; (2) the implementation of the action plan; and (3) the use of the web-based tool.

**Figure 2:**
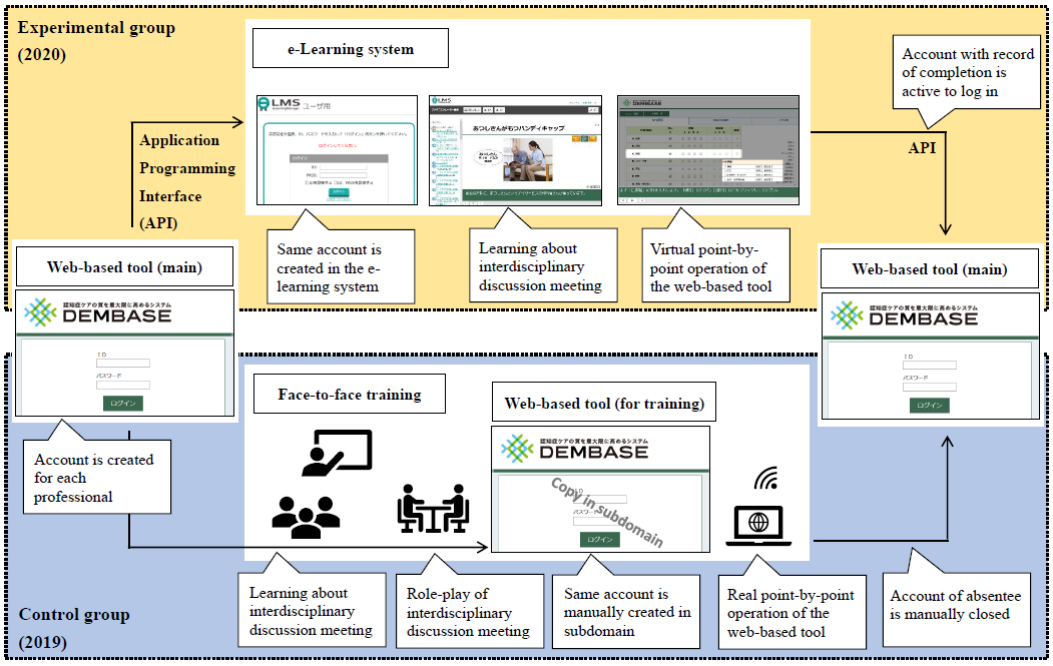
Web-based tool and training course of the program. DEMBASE: dementia behavior analysis and support enhancement.

The training course was based on the consideration of neuropsychiatric symptoms as communicating unmet needs [26-28]. Based on global evidence, psychosocial interventions including goal setting, such as providing pleasant activities, providing outdoor activities, and removal of environmental triggers, were recommended to address these unmet needs [29]. The web-based tool was explained to each participating professional during the training course (Figure 2). Further details regarding the training components and topics of discussion from debriefing meetings are reported elsewhere [23].

##### e-Learning Training Course and Web-Based Debriefing Meeting

The e-learning training course was delivered thrice to 298 professional caregivers between July and December 2020. Of the 298 caregivers, 268 (89.9%) completed the course. On average, professionals spent a total of 331.1 (SD 262.3) minutes to complete the course. The e-learning system was developed based on the framework provided by the Ginger App Company. The course included learning about interdisciplinary discussion meetings and virtual point-by-point operation of the web-based tool (Figure 2). Every text and direction on the screen was followed by voices that were developed using Amazon Polly (Amazon Web Services). The e-learning course was designed and developed by the Tokyo Metropolitan Institute of Medical Science, in collaboration with the face-to-face training course’s instructors and the Tokyo Metropolitan Government. A prototype of the course was completed by four professionals who had not participated in the face-to-face training. Based on their feedback, revisions were made to the course, after which it was finalized.

A debriefing meeting was delivered via a Zoom meeting (Zoom Video Communications) and divided into two parts. The first 90-minute debriefing meeting was within 4 weeks after the e-learning course was completed and was aimed at reviewing what participants had learned during the course, motivating use of the DEMBASE program, and setting a task by the next debriefing meeting. Hereafter, a 90-minute debriefing meeting was set 4 weeks later to share participant experiences related to the program. The first debriefing meetings were held 21 times between September 2020 and February 2021. The second debriefing meetings were held 14 times between November 2020 and March 2021. Both debriefing meetings were organized by instructors who had completed the face-to-face training course and the train-the-trainer program offered by the Tokyo Metropolitan Institute of Medical Science.

##### Face-to-Face Training Course and Debriefing Meeting

A 1-day, face-to-face training course was delivered 12 times to 274 professional caregivers between May 2019 and February 2020. Of the 274 caregivers who intended to participate in the course, 252 (92.0%) attended and completed it. The course included learning about, and role-play of, interdisciplinary discussion meetings and real point-by-point operation of the web-based tool (Figure 2).

Face-to-face, 3-hour debriefing meetings were held nine times, 4 to 6 weeks after the face-to-face training, which was conducted between July 2019 and February 2020. Debriefing meetings were organized by instructors who had completed the face-to-face training course and the train-the-trainer program, offered by the Tokyo Metropolitan Institute of Medical Science.

#### Interdisciplinary Discussion Meeting

Once the training course was completed, the professionals held an interdisciplinary discussion meeting with other care professionals to evaluate the neuropsychiatric symptoms of each participant with dementia, to specify their unmet needs, and to establish an interdisciplinary action plan to meet those needs. The medications prescribed to each participant with dementia were also assessed.

These components were included to promote a plan-do-study-act (PDSA) cycle, developed according to the team-based dementia case management model. The PDSA cycle is widely used as a quality improvement method in health care settings [30]. If the level of neuropsychiatric symptoms assessed was not reduced during follow-up evaluations, care professionals reviewed unmet needs and revised the action plan during the discussion meeting.

Unmet needs, content of the action plan, and types of caregivers involved in the meeting were categorized and recorded using the web-based tool. The categories of unmet needs were developed by referring to the findings on associations between neuropsychiatric symptoms and basic physical needs [31] as well as environmental sources of discomfort [32,33].

#### Web-Based Tool

The web-based tool provided a visualization of longitudinal changes in neuropsychiatric symptoms measured by the Neuropsychiatric Inventory (NPI) to inform interdisciplinary decision making. Professionals input the information collected during the discussion meeting into the web-based tool. The individual characteristics of persons with dementia were recorded at registration, including birth year and month, sex, and type of dementia. Participating professionals performed a baseline evaluation and follow-up evaluations to assess neuropsychiatric symptoms at each time point for each person with dementia until the end of the fiscal year.

#### Debriefing Meeting

The participating professionals attended a debriefing meeting after training where they were divided into groups of 4 to 6 members to share their experiences about the program.

### Measurement

The primary outcome measure for feasibility was the percentage of professionals with “full implementation” of the DEMBASE program. Full implementation was defined as the completion of a follow-up evaluation of neuropsychiatric symptoms for at least one person with dementia.

The primary outcome measure for efficacy was the change in the level of neuropsychiatric symptoms in persons with dementia who received full implementation of the program. The secondary outcome measure was the change in the percentage of psychotropic prescriptions. We collected this information on neuropsychiatric symptoms and prescribed medications, which was available via the web-based tool.

The NPI–Nursing Home version (NPI-NH) was used to assess the incidence, frequency, and severity of neuropsychiatric symptoms. The original NPI-NH was comprised of 12 items to rate the frequency and severity of neuropsychiatric symptoms in persons with dementia [34-37]. Scores for each item ranged from 0 to 12, with higher scores indicating more severe symptoms. Frequency and severity scores were multiplied to determine a total score ranging from 0 to 144. The Japanese version of the NPI-NH had good validity and reliability [38].

Drug names and daily dosage were recorded for each prescribed medication. In this study, the presence of prescribed medication for the nervous system was used for analysis based on the Anatomical Therapeutic Chemical classification system: percentages of the prescriptions were calculated for analgesics (N02), antipsychotics (N05A), anxiolytics (N05B), hypnotics and sedatives (N05C), antidepressants (N06A), and antidementia drugs (N06D).

### Statistical Analysis

The percentage of professionals who implemented follow-up evaluations was compared between the experimental and control groups using a chi-square test.

The change in the level of neuropsychiatric symptoms in persons with dementia from baseline to the most recent follow-up was compared between the two groups. Multiple linear regression analysis was used, including the NPI score at the most recent follow-up evaluation as a dependent variable and group as an independent variable. The NPI score at baseline was entered as a covariate.

The within-subject effect size was calculated using the Cohen formula for the NPI score. The effect size was assumed to be low if *d* values varied by approximately 0.20, medium if *d* values varied by approximately 0.50, and large if *d* values were greater than 0.80 [39,40].

Between-group differences were also examined for changes in psychotropic prescriptions using binomial logistic regression analysis. The model included the rate of prescription at the most recent follow-up evaluation as a dependent variable, group as an independent variable, and rate of prescription at baseline as a covariate.

Statistical significance was considered at an overall α value of .05. All statistical analyses were conducted using Stata software (version 16.1; StataCorp LLC).

### Ethics

This project was approved by the Ethics Review Board of the Tokyo Metropolitan Institute of Medical Science (No. 20-41) and Tohoku University (2021-1-293). This project was completed in accordance with the Helsinki Declaration of 1975 (as revised in 2013).

## Results

### Characteristics of Care Professionals

The experimental group (ie, e-learning) included professionals from in-home care management (131/268, 48.9%), in-home care services (93/268, 34.7%), and residential care services (44/268, 16.4%) (Table 2). The distribution of types of service did not significantly differ between the experimental group and the control group (ie, face-to-face) (*χ*^2^_2_=4.1, *P*=.13).

**Table 2:** Types of service of professional caregivers who completed the training course to use a web-based tool of psychosocial interventions for neuropsychiatric symptoms of dementia.

Type of service			Professionals in e-learning group, 2020 (n=268), n (%)		Professionals in face-to-face training group, 2019 (n=252), n (%)
**In-home care management**					
	Community general support center	50 (18.7)		8 (3.2)	
	Initial phase intensive support team	12 (4.5)		1 (0.4)	
	Care management agency	69 (25.7)		101 (40.1)	
**In-home care service**					
	Day care at center	41 (15.3)		52 (20.6)	
	Rehabilitation at center	3 (1.1)		2 (0.8)	
	Rehabilitation at home	2 (0.7)		0 (0)	
	Home-visiting nursing care	32 (11.9)		2 (0.8)	
	Personal care at home	10 (3.7)		16 (6.3)	
	In-home multiple service	5 (1.9)		11 (4.4)	
**Residential care service**					
	Group home	19 (7.1)		31 (12.3)	
	Housing with care	3 (1.1)		4 (1.6)	
	Geriatric intermediate care facility	4 (1.5)		3 (1.2)	
	Nursing home	17 (6.3)		21 (8.3)	
	Long-term sanatorium	1 (0.4)		0 (0)	

### Feasibility: Full Implementation

Of the 268 professionals who completed the e-learning course, 56 (20.9%) implemented follow-up evaluations of neuropsychiatric symptoms by the end of March 2021 (Table 3). The percentage of full implementation in the experimental group was significantly lower than that of professionals who completed the face-to-face training course in 2019.

**Table 3:** Percentage of professionals who implemented follow-up evaluations of neuropsychiatric symptoms.

Follow-up evaluation	Professionals in the e-learning group, 2020 (n=268), n (%)	Professionals in the face-to-face training group, 2019 (n=252)	Chi-square (*df*)	*P* value
Implemented	56 (20.9)	114 (45.2)	52.0 (1)	<.001
Did not implement	212 (79.1)	138 (54.8)	—^a^	—

^a^Chi-square and *P* values are reported in the top row of compared items.

### Characteristics of Persons With Dementia

There were 63 persons with dementia who received follow-up evaluations by professionals who completed the e-learning course (Table 4). The distribution of types of service did not significantly differ between the experimental group and the control group (ie, face-to-face) (*χ*^2^_2_=1.4, *P*=.51). There was no significant between-group difference in sex (*χ*^2^_2_=0.1, *P*=.81) or age (*t*_145.72_=0.42, *P*=.68).

Some people in the experimental group (n=63) were identified to have unmet needs at baseline related to the following: social isolation (n=30, 48%), sleepiness or tiredness (n=27, 43%), urination (n=27, 43%), hydration (n=27, 43%), and evacuation (n=24, 38%). Some people in the control group (n=132) presented with needs related to sleepiness or tiredness (n=65, 49.2%), evacuation (n=56, 42.4%), feeling uncomfortable (n=53, 40.2%), and pain (n=52, 39.4%) (Table S1 in Multimedia Appendix 1).

**Table 4:** Baseline characteristics of persons with full implementation of the psychosocial dementia care program in Tokyo.

Characteristic			Persons with dementia in e-learning group, 2020 (n=63), n (%)		Persons with dementia in face-to-face training group, 2019 (n=132), n (%)
**Age in years**					
	≤64	0 (0)		4 (3.0)	
	65-74	6 (9.5)		12 (9.1)	
	75-84	21 (33.3)		38 (28.8)	
	85-94	32 (50.8)		69 (52.3)	
	≥95	4 (6.3)		9 (6.8)	
**Sex**					
	Male	18 (28.6)		40 (30.3)	
	Female	45 (71.4)		92 (69.7)	
**In-home care management**					
	Community general support center	12 (19.0)		1 (0.8)	
	Initial phase intensive support team	1 (1.6)		0 (0)	
	Care management agency	16 (2.5)		49 (37.1)	
**In-home care**					
	Day care at center	8 (12.7)		36 (27.3)	
	Rehabilitation at center	3 (4.8)		0 (0)	
	Rehabilitation at home	1 (1.6)		0 (0)	
	Personal care at home	1 (1.6)		5 (3.8)	
	Home-visiting nursing care	7 (11.1)		2 (1.5)	
	In-home multiple service	3 (4.8)		9 (6.8)	
**Residential care**					
	Group home	3 (4.8)		14 (10.6)	
	Housing with care	0 (0)		4 (3.0)	
	Geriatric intermediate care facility	2 (3.2)		3 (2.3)	
	Nursing home	6 (9.5)		9 (6.8)	

### Efficacy: Neuropsychiatric Symptoms

The 63 persons with dementia in the experimental group had a mean NPI score of 20.4 (SD 16.2) at baseline. The symptoms that were frequently observed in the experimental group at baseline included agitation or aggression (n=48, 76%), anxiety (n=35, 56%), delusion (n=32, 51%), irritability or lability (n=30, 48%), and depression or dysphoria (n=26, 41%). These symptoms were also observed in the control group at baseline (Table S2 in Multimedia Appendix 1). The mean NPI score was significantly reduced to 14.3 (SD 13.4) at the most recent follow-up (paired *t*_62_=4.10, *P*<.001). The level of reduction was not significantly different from that in the control group (Table 5). The effect size was assumed to be medium for both the experimental group (*d*_rm [repeated measures]_=0.40) and the control group (*d*_rm_=0.36).

**Table 5:** Change in level of neuropsychiatric symptoms from baseline to the most recent follow-up evaluation.

Measure			Persons with dementia in e-learning group, 2020 (n=63)		Persons with dementia in face-to-face training group, 2019 (n=132)		Baseline-adjusted difference^a^		*P* value
**NPI score^b^, mean (SD)**									
	Baseline	20.4 (16.2)		23.8 (18.1)		N/A^c^		N/A	
	Follow-up	14.3 (13.4)		17.4 (17.2)		N/A		N/A	
	Difference	6.1 (11.8)		6.4 (10.6)		–0.61		.69	
Effect size: Cohen *d*_rm (repeated measures)_			0.40		0.36		N/A		N/A

^a^The baseline-adjusted difference was examined using multiple linear regression analysis with the Neuropsychiatric Inventory (NPI) score at baseline as a covariate.

^b^Levels of neuropsychiatric symptoms were assessed using the total NPI score, which ranged from 0 to 144.

^c^N/A: not applicable; these values were only calculated for the NPI score difference.

### Efficacy: Psychotropic Prescriptions

The percentage of psychotropic prescriptions was not significantly different between the experimental and control groups (Table 6).

**Table 6:** Change in psychotropic prescriptions from baseline to the most recent follow-up evaluation.

Prescription			Persons with dementia in e-learning group, 2020 (n=63), n (%)		Persons with dementia in face-to-face training group, 2019 (n=132), n (%)		Baseline-adjusted difference^a^		*P* value
**Analgesics**									
	Baseline	10 (15.9)		20 (15.2)		—^b^		—	
	Follow-up	9 (14.3)		19 (14.4)		0.68		.72	
**Antipsychotics**									
	Baseline	10 (15.9)		23 (17.4)		—		—	
	Follow-up	12 (19.0)		27 (20.5)		1.03		.97	
**Anxiolytics**									
	Baseline	7 (11.1)		7 (5.3)		—		—	
	Follow-up	7 (11.1)		7 (5.3)		1.52		.78	
**Hypnotics and sedatives**									
	Baseline	7 (11.1)		14 (10.6)		—		—	
	Follow-up	6 (9.5)		13 (9.8)		0.46		.61	
**Antidepressants**									
	Baseline	2 (3.2)		4 (3.0)		—		—	
	Follow-up	3 (4.8)		6 (4.5)		1.05		.97	
**Antidementia drugs**									
	Baseline	21 (33.3)		59 (44.7)		—		—	
	Follow-up	24 (38.1)		64 (48.5)		0.99		.99	

^a^The baseline-adjusted difference was examined using binomial logistic regression analysis with psychotropic prescription at baseline as a covariate.

^b^Baseline-adjusted differences and *P* values are reported in the bottom row of compared items.

## Discussion

### Overview

With an increasing demand for e-learning platforms for essential workers, it is vital that the feasibility and efficacy of the digitally transformed DEMBASE program be assessed. Thus, this study aimed to test the feasibility and efficacy of the e-learning training course, distributed to 268 professionals between July and December 2020 during the COVID-19 pandemic, by comparing it to the face-to-face training course and assessing which program more effectively addressed neuropsychiatric symptoms of dementia.

### Principal Findings

By replacing the face-to-face training course with the e-learning course, professionals who may not have participated in the program otherwise were provided the opportunity to participate in the DEMBASE program. This may explain the reduction in neuropsychiatric symptoms, which was significant with a medium effect size in this study. Similarly, the natural reduction of NPI scores was –0.8 over 6 months in a randomized controlled study [22]; thus, the program with e-learning training in this study appeared to be clinically effective. The efficacy of the program was not significantly different between the two groups. However, the percentage of full implementation was significantly lower among professionals who completed the e-learning course than among those who completed the face-to-face training course. Therefore, replacing face-to-face training courses with e-learning training courses appeared to be less effective in encouraging professionals to implement the program.

Compared to the face-to-face training course, the e-learning course may have lacked motivational triggers for implementation for professionals. Unlike e-learning, face-to-face training courses involved role-play in an interdisciplinary discussion meeting that may have yielded human interactions among participating professionals and between instructors and participants. Although we divided debriefing meetings into two parts and intended for the first meeting to offer interactions between participants, the 90-minute Zoom meeting may have been suboptimal to substitute the motivational triggers that emerged in the face-to-face training course. In addition, facilitating such an interaction can be complemented by using online communities, such as Facebook and LinkedIn; however, these online platforms are not often used by professional caregivers [41]. Therefore, the use of online communities in the DEMBASE program, which has not yet been introduced because of privacy concerns with social networks, is encouraged and should be further investigated.

The reduced implementation could also have been because of the increased workload of professionals due to the COVID-19 pandemic. This pandemic may have had a negative impact on the feasibility of the DEMBASE program among professionals. As preventive measures against COVID-19 were added to their daily practice, professionals had less time to implement the program. Furthermore, face-to-face contact was avoided, even for communication between professionals, under the lockdown and other public health and social measures that were implemented. In this study, we replaced a face-to-face debriefing meeting with a Zoom meeting, thus providing professionals with an opportunity to participant in a web-based meeting. Furthermore, we proposed to the participants that an interdisciplinary discussion meeting also be held via a Zoom meeting. Municipal governments also provided financial support to long-term care providers to refund the costs of purchasing tablet devices for program implementation. However, long-term care service providers generally have several barriers to implementing digital transformation in dementia care, including information technology infrastructure instability and a reluctance to change established practices and routines [42]. Thus, additional technical support is warranted to enable long-term care providers to have essential communication between professionals and with people with dementia amid the COVID-19 pandemic.

The reduced rate of full program implementation in the e-learning course may also be attributed to possible misdirection in the recruitment process phrased as “participation in e-learning course.” Some professionals confessed in the debriefing meeting that they recognized the DEMBASE program as an e-learning course rather than the PDSA cycle combining an interdisciplinary discussion meeting with a web-based tool. Therefore, a video message from “peer” professionals who have implemented the program and recognized its effectiveness may improve the readiness of applicants for participation. Such peer messages can also be integrated into the content of the e-learning training course. Additionally, recruiting two or more professionals from the same provider could encourage program implementation, as they may be able to commence the program with a small discussion meeting with those who have completed the course.

### Strengths and Limitations

A strength of this study was that the efficacy and feasibility of replacing a face-to-face course with an e-learning training course was tested using a quasi-experimental design, thus addressing a gap in the literature. A limitation was the lack of information on the characteristics of professionals. That is, differences in the gender and age of the professionals in the experimental (ie, e-learning) and control (ie, face-to-face) groups may have influenced the level of resistance to technology usage [43]. Based on the agreement for this study with the Tokyo Metropolitan Government, we were not allowed to access such information. In addition, the small number of persons with follow-up evaluations in the experimental group may have caused insufficient statistical power to examine the differences in changes in neuropsychiatric symptoms.

### Conclusions

The e-learning training course provided the opportunity for professionals to participate in the psychosocial dementia care program during the COVID-19 pandemic. Following completion of the e-learning course, the program sustained an equal level of efficacy in reducing neuropsychiatric symptoms compared to the face-to-face training course. However, the feasibility appeared to be suboptimal as the rate of implementation was low among professionals who completed the e-learning course. Further strategies to encourage implementation of the program should, thus, be developed to provide motivational triggers for implementation, such as distribution of a video message from peer professionals in the recruitment process and the e-learning training course as well as technical support for care professionals who work for people with dementia. Additionally, the use of online communities by the professionals in the program should be further investigated.

## Appendix

Multimedia Appendix 1 Unmet needs identified and presence of neuropsychiatric symptoms evaluated during the interdisciplinary discussion meeting at baseline.

## Abbreviations

DEMBASE: dementia behavior analysis and support enhancement
NPI: Neuropsychiatric Inventory
NPI-NH: Neuropsychiatric Inventory–Nursing Home version
PDSA: plan-do-study-act

## References

[1] Prince M, Bryce R, Albanese E, Wimo A, Ribeiro W, Ferri CP (2013). The global prevalence of dementia: A systematic review and metaanalysis. Alzheimers Dement.

[2] Prince M, Wimo A, Guerchet M, Ali GC, Wu YT (2015). World Alzheimer Report 2015. The Global Impact of Dementia: An Analysis of Prevalence, Incidence, Cost and Trends.

[3] Asada T, Matsuda H, Asada T, Tokumaru AM (2017). Epidemiology of dementia in Japan. Neuroimaging Diagnosis for Alzherimer's Disease and Other Dementias.

[4] Livingston G, Huntley J, Sommerlad A, Ames D, Ballard C, Banerjee S, Brayne C, Burns A, Cohen-Mansfield J, Cooper C, Costafreda SG, Dias A, Fox N, Gitlin LN, Howard R, Kales HC, Kivimäki M, Larson EB, Ogunniyi A, Orgeta V, Ritchie K, Rockwood K, Sampson EL, Samus Q, Schneider LS, Selbæk G, Teri L, Mukadam N (2020). Dementia prevention, intervention, and care: 2020 report of the Lancet Commission. Lancet.

[5] Verity R, Okell LC, Dorigatti I, Winskill P, Whittaker C, Imai N, Cuomo-Dannenburg G, Thompson H, Walker PGT, Fu H, Dighe A, Griffin JT, Baguelin M, Bhatia S, Boonyasiri A, Cori A, Cucunubá Z, FitzJohn R, Gaythorpe K, Green W, Hamlet A, Hinsley W, Laydon D, Nedjati-Gilani G, Riley S, van Elsland S, Volz E, Wang H, Wang Y, Xi X, Donnelly CA, Ghani AC, Ferguson NM (2020). Estimates of the severity of coronavirus disease 2019: A model-based analysis. Lancet Infect Dis.

[6] Yang J, Zheng Y, Gou X, Pu K, Chen Z, Guo Q, Ji R, Wang H, Wang Y, Zhou Y (2020). Prevalence of comorbidities and its effects in patients infected with SARS-CoV-2: A systematic review and meta-analysis. Int J Infect Dis.

[7] Guan W, Liang W, Zhao Y, Liang H, Chen Z, Li Y, Liu X, Chen R, Tang C, Wang T, Ou C, Li L, Chen P, Sang L, Wang W, Li J, Li C, Ou L, Cheng B, Xiong S, Ni Z, Xiang J, Hu Y, Liu L, Shan H, Lei C, Peng Y, Wei L, Liu Y, Hu Y, Peng P, Wang J, Liu J, Chen Z, Li G, Zheng Z, Qiu S, Luo J, Ye C, Zhu S, Cheng L, Ye F, Li S, Zheng J, Zhang N, Zhong N, He J, China Medical Treatment Expert Group for COVID-19 (2020). Comorbidity and its impact on 1590 patients with COVID-19 in China: A nationwide analysis. Eur Respir J.

[8] Bunn F, Burn A, Goodman C, Rait G, Norton S, Robinson L, Schoeman J, Brayne C (2014). Comorbidity and dementia: A scoping review of the literature. BMC Med.

[9] Wang H, Li T, Barbarino P, Gauthier S, Brodaty H, Molinuevo JL, Xie H, Sun Y, Yu E, Tang Y, Weidner W, Yu X (2020). Dementia care during COVID-19. Lancet.

[10] Simonetti A, Pais C, Jones M, Cipriani MC, Janiri D, Monti L, Landi F, Bernabei R, Liperoti R, Sani G (2020). Neuropsychiatric symptoms in elderly with dementia during COVID-19 pandemic: Definition, treatment, and future directions. Front Psychiatry.

[11] Lyketsos CG, Lopez O, Jones B, Fitzpatrick AL, Breitner J, DeKosky S (2002). Prevalence of neuropsychiatric symptoms in dementia and mild cognitive impairment: Results from the Cardiovascular Health Study. JAMA.

[12] Selbæk G, Engedal K, Bergh S (2013). The prevalence and course of neuropsychiatric symptoms in nursing home patients with dementia: A systematic review. J Am Med Dir Assoc.

[13] Maust DT, Langa KM, Blow FC, Kales HC (2017). Psychotropic use and associated neuropsychiatric symptoms among patients with dementia in the USA. Int J Geriatr Psychiatry.

[14] Bränsvik V, Granvik E, Minthon L, Nordström P, Nägga K (2021). Mortality in patients with behavioural and psychological symptoms of dementia: A registry-based study. Aging Ment Health.

[15] Tao Y, Peters ME, Drye LT, Devanand DP, Mintzer JE, Pollock BG, Porsteinsson AP, Rosenberg PB, Schneider LS, Shade DM, Weintraub D, Yesavage J, Lyketsos CG, Munro CA (2018). Sex differences in the neuropsychiatric symptoms of patients with Alzheimer's disease. Am J Alzheimers Dis Other Demen.

[16] Numbers K, Brodaty H (2021). The effects of the COVID-19 pandemic on people with dementia. Nat Rev Neurol.

[17] Borelli WV, Augustin MC, de Oliveira PBF, Reggiani LC, Bandeira-de-Mello RG, Schumacher-Schuh AF, Chaves MLF, Castilhos RM (2021). Neuropsychiatric symptoms in patients with dementia associated with increased psychological distress in caregivers during the COVID-19 pandemic. J Alzheimers Dis.

[18] El Haj M, Altintas E, Chapelet G, Kapogiannis D, Gallouj K (2020). High depression and anxiety in people with Alzheimer's disease living in retirement homes during the COVID-19 crisis. Psychiatry Res.

[19] Pongan E, Dorey J, Borg C, Getenet JC, Bachelet R, Lourioux C, Laurent B, Rey R, Rouch I (2021). COVID-19: Association between increase of behavioral and psychological symptoms of dementia during lockdown and caregivers’ poor mental health. J Alzheimers Dis.

[20] Manini A, Brambilla M, Maggiore L, Pomati S, Pantoni L (2021). The impact of lockdown during SARS-CoV-2 outbreak on behavioral and psychological symptoms of dementia. Neurol Sci.

[21] Leontjevas R, Knippenberg IAH, Smalbrugge M, Plouvier AOA, Teunisse S, Bakker C, Koopmans RTCM, Gerritsen DL (2021). Challenging behavior of nursing home residents during COVID-19 measures in the Netherlands. Aging Ment Health.

[22] Nakanishi M, Endo K, Hirooka K, Granvik E, Minthon L, Nägga K, Nishida A (2018). Psychosocial behaviour management programme for home-dwelling people with dementia: A cluster-randomized controlled trial. Int J Geriatr Psychiatry.

[23] Nakanishi M, Ziylan C, Bakker T, Granvik E, Nägga K, Nishida A (2021). Facilitators and barriers associated with the implementation of a Swedish psychosocial dementia care programme in Japan: A secondary analysis of qualitative and quantitative data. Scand J Caring Sci.

[24] Faul F, Erdfelder E, Lang A, Buchner A (2007). G*Power 3: A flexible statistical power analysis program for the social, behavioral, and biomedical sciences. Behav Res Methods.

[25] Faul F, Erdfelder E, Buchner A, Lang A (2009). Statistical power analyses using G*Power 3.1: Tests for correlation and regression analyses. Behav Res Methods.

[26] Algase DL, Beck C, Kolanowski A, Whall A, Berent S, Richards K, Beattie E (2016). Need-driven dementia-compromised behavior: An alternative view of disruptive behavior. Am J Alzheimers Dis Other Demen.

[27] Cohen-Mansfield J, Werner P (2016). Environmental influences on agitation: An integrative summary of an observational study. Am J Alzheimers Care Relat Disord Res.

[28] Kovach CR, Noonan PE, Schlidt AM, Wells T (2005). A model of consequences of need-driven, dementia-compromised behavior. J Nurs Scholarsh.

[29] Lord K, Beresford-Dent J, Rapaport P, Burton A, Leverton M, Walters K, Lang I, Downs M, Manthorpe J, Boex S, Jackson J, Ogden M, Cooper C (2020). Developing the New Interventions for independence in Dementia Study (NIDUS) theoretical model for supporting people to live well with dementia at home for longer: A systematic review of theoretical models and randomised controlled trial evidence. Soc Psychiatry Psychiatr Epidemiol.

[30] Taylor MJ, McNicholas C, Nicolay C, Darzi A, Bell D, Reed JE (2014). Systematic review of the application of the plan-do-study-act method to improve quality in healthcare. BMJ Qual Saf.

[31] Bédard A, Landreville P, Voyer P, Verreault R, Vézina J (2011). Reducing verbal agitation in people with dementia: Evaluation of an intervention based on the satisfaction of basic needs. Aging Ment Health.

[32] Cohen-Mansfield J, Thein K, Marx MS, Dakheel-Ali M, Jensen B (2013). Sources of discomfort in persons with dementia. JAMA Intern Med.

[33] Cohen-Mansfield J, Thein K, Marx MS, Dakheel-Ali M, Jensen B (2015). Sources of discomfort in persons with dementia: Scale and initial results. Behav Neurol.

[34] Cummings JL, Mega M, Gray K, Rosenberg-Thompson S, Carusi DA, Gornbein J (1994). The Neuropsychiatric Inventory: Comprehensive assessment of psychopathology in dementia. Neurology.

[35] Cummings JL (1997). The Neuropsychiatric Inventory: Assessing psychopathology in dementia patients. Neurology.

[36] Kaufer D, Cummings J, Christine D, Bray T, Castellon S, Masterman D, MacMillan A, Ketchel P, DeKosky ST (1998). Assessing the impact of neuropsychiatric symptoms in Alzheimer's disease: The Neuropsychiatric Inventory Caregiver Distress Scale. J Am Geriatr Soc.

[37] Wood S, Cummings JL, Hsu M, Barclay T, Wheatley MV, Yarema KT, Schnelle JF (2000). The use of the neuropsychiatric inventory in nursing home residents. Characterization and measurement. Am J Geriatr Psychiatry.

[38] Selbæk G, Kirkevold Ø, Sommer OH, Engedal K (2007). The reliability and validity of the Norwegian version of the Neuropsychiatric Inventory, Nursing Home Version (NPI-NH). Int Psychogeriatr.

[39] Cohen J (1988). Statistical Power Analysis for the Behavioral Sciences. 2nd edition.

[40] Lakens D (2013). Calculating and reporting effect sizes to facilitate cumulative science: A practical primer for t-tests and ANOVAs. Front Psychol.

[41] Hattink B, Meiland F, van der Roest H, Kevern P, Abiuso F, Bengtsson J, Giuliano A, Duca A, Sanders J, Basnett F, Nugent C, Kingston P, Dröes RM (2015). Web-based STAR e-learning course increases empathy and understanding in dementia caregivers: Results from a randomized controlled trial in the Netherlands and the United Kingdom. J Med Internet Res.

[42] Dugstad J, Eide T, Nilsen ER, Eide H (2019). Towards successful digital transformation through co-creation: A longitudinal study of a four-year implementation of digital monitoring technology in residential care for persons with dementia. BMC Health Serv Res.

[43] Baudin K, Gustafsson C, Frennert S (2020). Views of Swedish elder care personnel on ongoing digital transformation: Cross-sectional study. J Med Internet Res.

