# Supplementary material for: e-Learning and Web-Based Tools for Psychosocial Interventions Addressing Neuropsychiatric Symptoms of Dementia During the COVID-19 Pandemic in Tokyo, Japan: Quasi-Experimental Study
Source: JMIR Med Educ. 2021 Oct 12;7(4):e30652. doi: 10.2196/30652 (PMC8513743; doi:10.2196/30652)
Supplement: Multimedia Appendix 1 [file mededu_v7i4e30652_app1.docx]

**Table S1.** Unmet needs identified during the interdisciplinary discussion meeting at baseline.

| N (%) at baseline | Experimental | Control |
| --- | --- | --- |
| Another person is bothering the person | 20 (31.7) | 43 (32.6) |
| Sleepiness or tiredness | 27 (42.9) | 65 (49.2) |
| Pain | 20 (31.7) | 52 (39.4) |
| Urination | 27 (42.9) | 46 (34.8) |
| Feeling uncomfortable | 14 (22.2) | 53 (40.2) |
| Evacuation | 24 (38.1) | 56 (42.4) |
| Rash/fungus | 15 (23.8) | 26 (19.7) |
| Blood pressure | 15 (23.8) | 33 (25.0) |
| Being cold/hot | 13 (20.6) | 35 (26.5) |
| Social interactions | 30 (47.6) | 45 (34.1) |
| Hearing | 11 (17.5) | 24 (18.2) |
| Medication | 17 (27.0) | 36 (27.3) |
| Meal | 20 (31.7) | 33 (25.0) |
| Hydration | 27 (42.9) | 46 (34.8) |
| Eyesight | 12 (19.0) | 26 (19.7) |
| Body positioning | 10 (15.9) | 25 (18.9) |
| Breathing | 4 (6.3) | 7 (5.3) |
| Lighting | 11 (17.5) | 15 (11.4) |
| Blood sugar | 5 (7.9) | 7 (5.3) |
| Furniture | 7 (11.1) | 14 (10.6) |
| Temperature | 3 (4.8) | 3 (2.3) |
| Pulse | 1 (1.6) | 10 (7.6) |
| Restraints | 1 (1.6) | 4 (3.0) |

**Table S2.** Presence of neuropsychiatric symptoms evaluated in the interdisciplinary discussion meeting at baseline.

| N (%) at baseline | Experimental | Control |
| --- | --- | --- |
| Delusion | 32 (50.8) | 58 (43.9) |
| Hallucination | 20 (31.7) | 33 (25.0) |
| Agitation/aggression | 48 (76.2) | 93 (70.5) |
| Depression/dysphoria | 26 (41.3) | 75 (56.8) |
| Anxiety | 35 (55.6) | 80 (60.6) |
| Elation/euphoria | 7 (11.1) | 30 (22.7) |
| Apathy/indifference | 25 (39.7) | 58 (43.9) |
| Disinhibition | 13 (21.6) | 39 (29.5) |
| Irritability/lability | 30 (47.6) | 62 (47.0) |
| Aberrant motor behavior | 23 (36.5) | 37 (28.0) |
| Sleep and nighttime behavior disorders | 17 (27.0) | 46 (34.8) |
| Appetite and eating changes | 14 (22.2) | 50 (37.9) |
